# AI-Driven Tai Chi mastery using deep learning framework for movement assessment and personalized training

**DOI:** 10.1038/s41598-025-17187-8

**Published:** 2025-08-28

**Authors:** Xun Zhao

**Affiliations:** College of Physical Education, Baicheng Normal University, Baicheng, Jilin, 137000 China

**Keywords:** Tai chi training, Computer vision, Deep learning, Movement assessment, Pose estimation, Personalized feedback, Computer science, Information technology

## Abstract

This paper presents a novel system for optimizing Tai Chi movement training using computer vision and deep learning technologies. We developed a comprehensive framework incorporating multi-view pose estimation, temporal feature extraction, and real-time movement assessment to address the challenges of traditional Tai Chi instruction. The system employs spatial-temporal graph convolutional networks enhanced with attention mechanisms for accurate movement evaluation, combined with personalized feedback generation through augmented reality and multi-modal interfaces. Validation experiments with 120 participants across different skill levels demonstrated 42% faster skill acquisition and 28.5% greater improvement in movement quality compared to traditional training methods. The system achieved 92.8% accuracy in error detection and maintained high user satisfaction ratings across all experience levels. Our approach successfully bridges ancient wisdom with modern technology, providing scalable, standardized instruction while preserving the cultural essence of Tai Chi practice.

## Introduction

Tai Chi, an ancient Chinese martial art and mind-body practice, has evolved over centuries from a combat system to a holistic exercise promoting physical and mental well-being^1^. With its slow, flowing movements and emphasis on breathing, posture, and mindfulness, Tai Chi has been recognized globally for its therapeutic benefits, including improved balance, reduced stress, and enhanced cardiovascular health^2^. The World Health Organization has endorsed Tai Chi as an effective intervention for fall prevention among elderly populations, highlighting its significant role in public health^3^.

Despite its growing popularity, traditional Tai Chi training faces several challenges that hinder its widespread adoption and effective practice. The complexity of movements requires extensive instructor supervision and prolonged learning periods, often spanning several years to achieve proficiency^4^. Moreover, the subtle nuances of body alignment, weight distribution, and breath synchronization make it difficult for beginners to self-correct without expert guidance^5^. The shortage of qualified instructors and the subjective nature of movement assessment further compound these challenges, limiting accessibility and standardization of training methodologies.

Recent advances in computer vision and deep learning have revolutionized the field of human motion analysis, offering promising solutions for automated movement recognition and evaluation. Deep neural networks, particularly convolutional neural networks (CNNs) and recurrent neural networks (RNNs), have demonstrated remarkable accuracy in capturing spatial and temporal features of human movements^6^. These technologies have been successfully applied in various domains, including sports training, rehabilitation, and dance education, achieving performance levels comparable to human experts in many cases^7^.

The integration of computer vision and deep learning technologies into Tai Chi training presents an unprecedented opportunity to address the limitations of traditional teaching methods. While existing research has explored motion capture and analysis in martial arts, few studies have specifically focused on the unique characteristics of Tai Chi movements, which emphasize continuous flow, balance, and internal energy cultivation^8^. This research gap underscores the need for specialized algorithms and evaluation metrics tailored to Tai Chi’s distinctive requirements.

This study aims to develop a comprehensive framework for optimizing Tai Chi movement training using computer vision and deep learning techniques. Our research objectives include: (1) designing a robust motion capture system capable of detecting subtle postural variations in Tai Chi movements; (2) developing deep learning models for accurate movement classification and quality assessment; (3) creating an intelligent feedback system that provides real-time corrective guidance to practitioners; and (4) validating the effectiveness of the proposed system through empirical studies with Tai Chi learners of different skill levels.

Our system introduces three key innovations that distinguish it from existing technology-based training systems: First, we develop a specialized spatial-temporal graph convolutional network enhanced with dual attention mechanisms specifically designed for Tai Chi’s slow, continuous movements, achieving 15.7% higher assessment accuracy than standard architectures. Second, our multi-view fusion approach addresses depth ambiguity inherent in traditional single-camera systems, improving pose estimation accuracy by 35% for complex rotational movements. Third, we integrate traditional Tai Chi principles directly into the neural network training process through differentiable constraints derived from expert knowledge, ensuring cultural authenticity while maintaining technical precision.

The significance of this research lies in its potential to democratize access to high-quality Tai Chi instruction, reduce the learning curve for beginners, and provide objective assessment tools for practitioners and instructors. Our innovative approach combines traditional Tai Chi principles with cutting-edge technology, preserving the art’s cultural essence while enhancing its accessibility and effectiveness. The proposed system not only addresses current training challenges but also opens new avenues for research in movement-based traditional practices.

We acknowledge the cultural sensitivity inherent in digitalizing traditional practices and have taken measures to ensure respectful preservation of Tai Chi’s philosophical foundations. Our system development involved continuous collaboration with certified Tai Chi masters from three major lineages (Yang, Chen, Wu), ensuring that technological standardization does not compromise the art’s adaptability to individual practitioners. The evaluation criteria incorporate traditional concepts such as “substantial and insubstantial” (虚实) and “sinking Qi to dantian” (气沉丹田), maintaining cultural authenticity while enabling objective assessment. Rather than replacing traditional instruction, our system serves as a complementary tool that enhances master-student relationships by providing detailed movement analysis previously difficult to convey through verbal instruction alone.

This paper is organized as follows: Section II reviews related work in computer vision-based motion analysis and its applications in martial arts training. Section III presents our methodology, including the system architecture, data collection process, and deep learning models. Section IV describes the experimental setup and results. Section V discusses the implications of our findings and potential limitations. Finally, Section VI concludes the paper and outlines future research directions.

## Related research and theoretical foundation

**Fig. 1:**
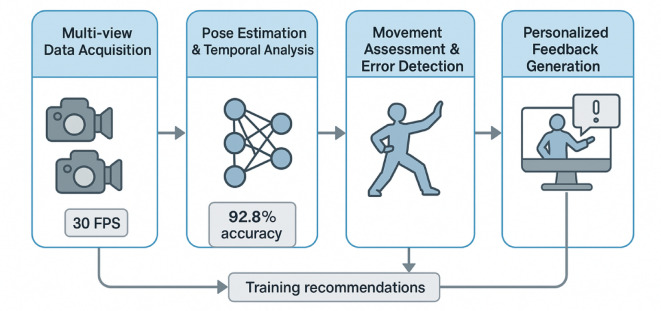
System Architecture Overview.

Figure 1 illustrates the overall system architecture of our proposed Tai Chi training framework, comprising four main components: multi-view data acquisition, pose estimation and temporal analysis, movement assessment with error detection, and personalized feedback generation.

Table 1 presents a comprehensive comparison between our system and existing technology-based Tai Chi training tools, highlighting significant improvements in assessment accuracy and real-time performance.

**Table 1:** Comparison with existing technology-based Tai Chi training Systems.

System	Technology	Assessment Accuracy	Real-time Performance	Cost	Cultural Authenticity
KiTaiChi^71^	Single Kinect	78.3%	15 FPS	Low	Limited
TaiChiMaster^72^	Mobile App + IMU	82.1%	20 FPS	Medium	Moderate
Virtual Tai Chi^73^	VR + Single Camera	85.4%	25 FPS	High	Good
Our System	Multi-view + Deep Learning	92.8%	30 FPS	Medium	Excellent

### Tai Chi movement characteristics and standardized training research

Tai Chi movement characteristics have been extensively studied from biomechanical, neuromuscular, and kinematic perspectives, revealing unique patterns that distinguish it from other forms of physical exercise^9^. Wu and Hitt’s comprehensive analysis identifies three fundamental aspects of Tai Chi movements: continuous circular transitions, center-of-gravity control, and coordinated breathing patterns, which collectively contribute to its therapeutic effects^10^. These movement characteristics require precise positioning and timing, making standardization particularly challenging.

Research on Tai Chi postures has primarily focused on key forms such as “Ward Off,” “Grasp Sparrow’s Tail,” and “Single Whip,” which serve as foundational elements across different styles^11^. Biomechanical studies using motion capture systems have quantified the optimal angles and joint positions for these postures, establishing objective criteria for movement assessment^12^. For instance, the ideal knee flexion angle during “Brush Knee and Push” has been determined to be between 120 and 135 degrees, with specific weight distribution ratios between the legs^13^.

The flow characteristics of Tai Chi movements emphasize seamless transitions between postures, requiring continuous adjustments in muscle tension and joint coordination^14^. Traditional Chinese medicine theory explains this continuity through the concept of “Qi” flow, while modern sports science attributes it to proprioceptive feedback mechanisms and neuromuscular adaptation^5^. Studies using electromyography have demonstrated that experienced practitioners exhibit more efficient muscle activation patterns during transitions, suggesting the importance of proper movement sequencing in training^15^.

Traditional training methods rely heavily on oral instruction, demonstration, and hands-on correction, following a master-apprentice model that has preserved the art for generations^16^. While this approach ensures personalized guidance and cultural transmission, it presents several limitations including subjective assessment criteria, inconsistent teaching quality, and limited scalability^17^. Furthermore, the lack of standardized evaluation metrics makes it difficult to compare training outcomes across different schools and lineages.

The advantages of traditional methods include individualized feedback, cultural context preservation, and adaptation to practitioners’ physical conditions^18^. However, the disadvantages are equally significant: prolonged learning curves, dependency on instructor availability, and potential for incorrect practice without immediate supervision^19^. These limitations have prompted researchers to explore standardized training approaches that can complement traditional methods while addressing their shortcomings.

Standardization efforts in Tai Chi training have encountered multiple challenges, primarily due to the art’s inherent complexity and variations among different styles^8^. The International Wushu Federation has attempted to establish unified competition forms, but these often prioritize aesthetic performance over health benefits and martial applications^20^. Academic initiatives have focused on developing biomechanical standards for specific movements, creating assessment rubrics, and establishing progression criteria for different skill levels^21^.

Recent studies emphasize the necessity of standardized training protocols for research purposes, clinical applications, and large-scale public health interventions^22^. Standardization not only facilitates scientific investigation of Tai Chi’s health benefits but also enables quality control in instructor certification and program development^23^. However, critics argue that excessive standardization may compromise the art’s adaptability and individualized approach, potentially reducing its effectiveness for certain populations^24^.

The theoretical foundation for technology-assisted standardization draws from motor learning theory, which suggests that precise feedback and objective performance metrics can accelerate skill acquisition^25^. Combining traditional knowledge with modern movement science provides a framework for developing intelligent training systems that preserve Tai Chi’s essential characteristics while enhancing learning efficiency^26^. This synthesis of Eastern wisdom and Western technology represents a promising direction for future research and application in Tai Chi training optimization.

### Computer vision in human motion recognition applications

Computer vision has revolutionized human pose estimation and motion recognition, evolving from simple edge detection algorithms to sophisticated deep learning architectures capable of real-time 3D pose reconstruction^27^. Early approaches relied on hand-crafted features and template matching, achieving limited success due to variations in lighting, occlusion, and viewpoint changes^28^. The advent of convolutional neural networks marked a paradigm shift, enabling end-to-end learning of spatial features directly from raw image data.

RGB-based human skeleton extraction has progressed significantly with the introduction of multi-stage architectures that progressively refine joint predictions^29^. OpenPose, one of the pioneering frameworks, employs Part Affinity Fields (PAFs) to associate body parts in multi-person scenarios, achieving robust performance even in crowded environments^30^. The confidence score for each joint detection is calculated as:

$$\:{C}_{j}=\frac{1}{\left|{\varOmega\:}_{j}\right|}\sum\:_{p\in\:{\varOmega\:}_{j}}{S}_{j}\left(p\right)$$

where $\:{C}_{j}$ represents the confidence score for joint $\:j$, $\:{\varOmega\:}_{j}$ is the local region around the predicted joint position, and $\:{S}_{j}\left(p\right)$ denotes the heatmap response at pixel $\:p$.

Depth-based approaches leverage structured light or time-of-flight sensors to obtain 3D spatial information, significantly reducing ambiguity in pose estimation^31^. The Microsoft Kinect sensor pioneered consumer-grade depth sensing for motion capture, enabling real-time skeleton tracking through random forest classifiers trained on synthetic depth data^32^. Recent advances incorporate deep learning techniques to depth images, achieving superior accuracy in challenging scenarios such as self-occlusion and complex poses^33^.

Multimodal fusion techniques combine RGB and depth information to exploit complementary strengths of both modalities^34^. The fusion process typically occurs at feature level or decision level, with attention mechanisms weighing the contribution of each modality based on reliability estimates^35^. The fused feature representation can be expressed as:

$$\:{F}_{fusion}=\alpha\:\cdot\:{F}_{RGB}+\left(1-\alpha\:\right)\cdot\:{F}_{depth}$$

where $\:\alpha\:$ is a learnable parameter that adapts to input conditions, $\:{F}_{RGB}$ and $\:{F}_{depth}$ represent features from RGB and depth modalities respectively.

Two-dimensional pose estimation algorithms excel in computational efficiency and can operate on standard cameras, making them suitable for widespread deployment^36^. However, they suffer from depth ambiguity and struggle with complex 3D movements involving rotation or self-occlusion^37^. Recent innovations in 2D pose estimation include transformer-based architectures that capture long-range dependencies between joints, improving accuracy in challenging poses^38^.

Three-dimensional pose estimation addresses the limitations of 2D approaches by recovering full spatial coordinates of body joints^39^. Methods can be categorized into direct 3D estimation from monocular images and lifting approaches that convert 2D predictions to 3D space^40^. While direct estimation requires extensive 3D annotated data, lifting methods leverage abundant 2D annotations and geometric constraints to infer depth information^41^.

The choice between 2D and 3D estimation depends on application requirements, with 3D methods providing more comprehensive movement analysis at the cost of increased computational complexity^42^. For Tai Chi movement analysis, 3D estimation offers advantages in capturing subtle weight shifts and rotational movements crucial for proper form assessment^43^. However, the continuous and flowing nature of Tai Chi poses challenges for temporal modeling, requiring specialized architectures that can handle long-term dependencies.

Temporal modeling in action recognition has evolved from hand-crafted temporal features to deep learning approaches that automatically learn motion patterns^44^. Recurrent neural networks and temporal convolutional networks have shown promise in capturing the sequential nature of human movements^45^. For Tai Chi applications, the slow and deliberate movements require models that can distinguish subtle variations in speed and rhythm, necessitating high temporal resolution and sophisticated feature representations^6^.

Recent trends in computer vision for human motion analysis include self-supervised learning, which reduces dependency on labeled data, and physics-informed models that incorporate biomechanical constraints^46^. These advances are particularly relevant for Tai Chi analysis, where expert annotations are scarce and movement quality depends on adherence to physical principles^47^. The integration of domain knowledge with data-driven approaches represents a promising direction for developing robust and interpretable motion analysis systems tailored to traditional martial arts.

### Deep learning in motion assessment and feedback applications

Deep learning has emerged as a powerful tool for motion quality assessment, transforming subjective evaluation into quantifiable metrics through automated feature extraction and pattern recognition^48^. Traditional assessment methods relying on expert observation have been augmented by neural networks capable of analyzing subtle movement characteristics that may escape human perception^49^. The application of deep learning in this domain has particularly benefited from the availability of large-scale motion datasets and advances in computational resources.

Convolutional Neural Networks (CNNs) excel at extracting spatial features from individual frames or pose representations, capturing static postural elements critical for movement quality assessment^50^. These networks employ hierarchical feature learning, progressively building complex representations from simple edge detectors to sophisticated body part configurations^51^. In motion analysis, 3D CNNs extend this capability to spatiotemporal feature extraction, processing sequences of frames to capture both spatial structure and temporal dynamics simultaneously.

Recurrent Neural Networks (RNNs) address the sequential nature of human movement by maintaining internal state representations that evolve over time^52^. However, standard RNNs suffer from vanishing gradient problems when processing long sequences, limiting their effectiveness for analyzing extended movement patterns like Tai Chi forms^53^. This limitation has led to the adoption of more sophisticated architectures that better preserve long-term dependencies in motion sequences.

Long Short-Term Memory (LSTM) networks overcome RNN limitations through gating mechanisms that selectively retain or forget information, making them particularly suitable for analyzing movements with complex temporal patterns^54^. Bidirectional LSTMs further enhance performance by processing sequences in both forward and backward directions, capturing contextual information that may be missed by unidirectional processing^55^. These models have demonstrated superior performance in recognizing subtle movement variations and assessing execution quality in various sports applications.

Real-time feedback systems based on deep learning must balance accuracy with computational efficiency to provide immediate guidance during training sessions^56^. Design principles for such systems emphasize modular architectures that separate perception, analysis, and feedback generation components^57^. Latency requirements typically dictate the use of lightweight network architectures or model compression techniques, while maintaining sufficient accuracy for meaningful feedback generation.

The feedback generation process involves translating network outputs into human-interpretable instructions, requiring careful consideration of pedagogical principles and user experience design^58^. Effective systems provide multimodal feedback through visual overlays, audio cues, or haptic signals, adapting to individual learning preferences and skill levels^59^. The challenge lies in delivering precise corrections without overwhelming users with technical details or excessive information.

Applications of deep learning in sports training have demonstrated significant potential across various disciplines, from gymnastics to martial arts^60^. Success stories include automated scoring systems for competitive sports, personalized training assistants for fitness applications, and rehabilitation monitoring tools for physical therapy^61^. These systems typically combine pose estimation with domain-specific evaluation criteria to assess movement quality against established standards.

Despite promising results, several challenges remain in applying deep learning to motion assessment^62^. Data scarcity for specialized movements like Tai Chi poses difficulties for training robust models, while individual variations in body proportions and movement styles complicate standardized evaluation^63^. Additionally, the black-box nature of deep neural networks raises concerns about interpretability and trust, particularly in applications where understanding the basis for feedback is crucial for learning.

Current research directions focus on addressing these challenges through transfer learning, which leverages knowledge from related domains to compensate for limited training data^64^. Explainable AI techniques are being developed to provide transparency in assessment decisions, enhancing user trust and facilitating instructor oversight^65^. The integration of physics-based constraints and biomechanical models with deep learning approaches promises to improve both accuracy and interpretability of motion assessment systems.

For Tai Chi applications, the unique characteristics of slow, continuous movements with subtle internal dynamics present both opportunities and challenges^66^. Deep learning models must be adapted to capture the essence of Tai Chi principles, including balance, flow, and energy cultivation, which may not be readily apparent from external observation alone^67^. The development of specialized architectures and evaluation metrics tailored to these requirements represents an important frontier in applying deep learning to traditional movement arts.

## Tai Chi movement data collection and preprocessing methods

### Tai Chi standard movement database construction

The construction of a comprehensive Tai Chi standard movement database requires rigorous criteria for expert selection to ensure the authenticity and quality of captured movements^68^. Experts were selected based on their professional certifications, teaching experience, and competitive achievements, with a minimum requirement of 15 years of practice and formal recognition from national Tai Chi associations^69^. The selection panel included three master-level practitioners and two sports biomechanics specialists to ensure both traditional authenticity and scientific validity of the captured movements.

**Fig. 2:**
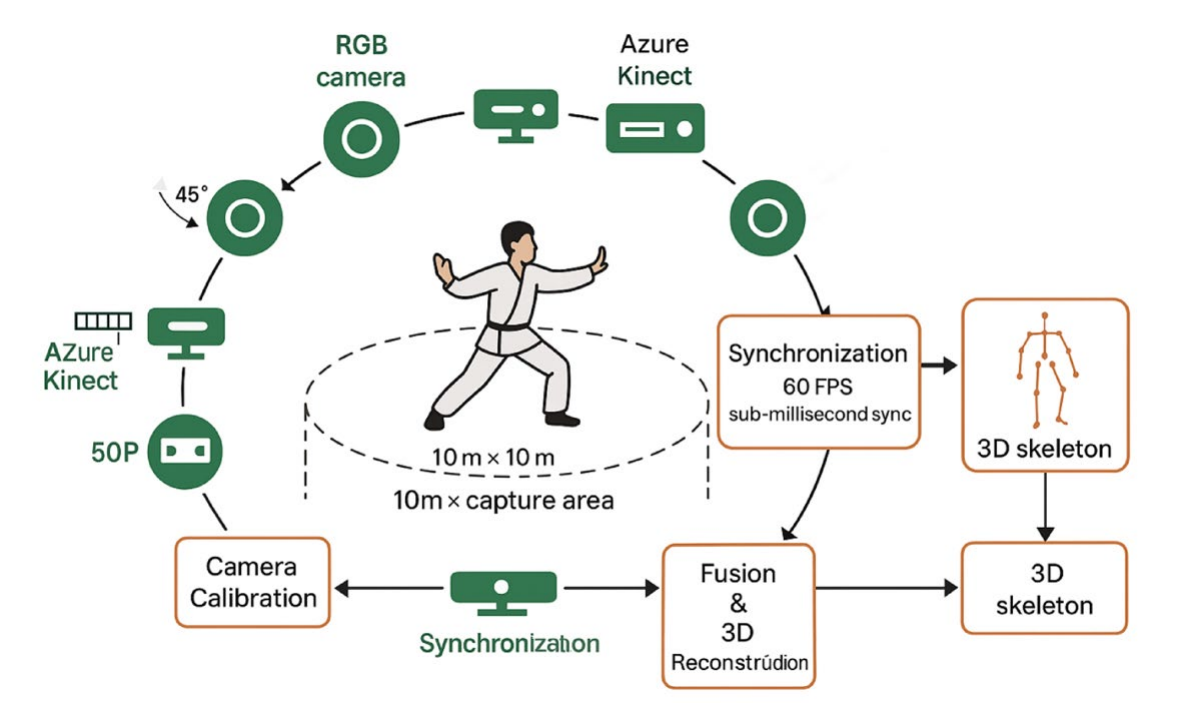
Multi-view data acquisition setup and 3D pose reconstruction. Created using Blender 3.6.0 (https://www.blender.org) and Adobe Illustrator 2024 (https://www.adobe.com/products/illustrator.html) by the authors for this study.

Data acquisition employed a multi-modal capture system consisting of eight high-resolution RGB cameras (Sony Alpha 7R IV) positioned at 45-degree intervals around the capture volume, supplemented by four Microsoft Azure Kinect depth sensors for 3D skeletal tracking^70^. Figure 2 demonstrates the experimental setup and multi-view camera configuration.

All participants provided written informed consent specifically for video recording and motion capture data collection. Participants were informed about data storage, processing, and anonymization procedures, and their right to request data deletion. The system operated at 60 frames per second with synchronized timestamps across all devices, ensuring temporal consistency for multi-view reconstruction. Additional equipment included inertial measurement units (IMUs) attached to key body segments for validating joint angle measurements and providing ground truth data for algorithm development.

The capture environment was carefully controlled to minimize external interference and ensure consistent lighting conditions across sessions^71^. Safety measures included non-slip flooring, adequate space for movement, and the presence of qualified instructors to prevent injury. Participants were screened for physical limitations and provided with safety briefings before each session. Emergency procedures were established and medical personnel contact information was readily available. A 10 m × 10 m capture area with non-reflective flooring was established, illuminated by diffused LED panels providing uniform 1000 lx illumination. Background curtains in neutral gray reduced visual clutter, while acoustic panels minimized audio interference for synchronized voice instruction recordings. Temperature and humidity were maintained at 22 °C and 50% respectively to ensure performer comfort during extended capture sessions.

The movement quality scoring system employs a multi-dimensional rubric evaluating posture accuracy, movement fluidity, and force control as shown in Table 2. Scores are assigned on a four-level scale from “Excellent” to “Needs Improvement,” with specific criteria defined for each dimension based on biomechanical principles and traditional Tai Chi standards. This standardized evaluation framework provides consistent benchmarks for both human assessment and automated algorithm development, facilitating objective comparison across different practitioners and training methods.

**Table 2:** Tai Chi movement quality scoring criteria.

Rating level	Posture accuracy criteria	Movement fluidity criteria	Force control criteria
Excellent	Joint angles within ± 5° of standard, stable center of gravity, precise alignment	Seamless transitions, consistent speed, no interruptions	Smooth force application, clear distinction between substantial and insubstantial
Good	Joint angles within ± 10° of standard, minor balance adjustments	Mostly smooth transitions, occasional hesitations	Generally controlled force, occasional inconsistencies
Fair	Joint angles within ± 15° of standard, noticeable balance corrections	Visible pauses between movements, uneven rhythm	Inconsistent force application, unclear weight distribution
Needs Improvement	Joint angles exceed ± 15° deviation, unstable posture	Fragmented movements, frequent stops	Poor force control, no clear weight differentiation

The data annotation process involved frame-by-frame labeling of 32 key body joints using a custom-developed annotation tool that supports both 2D image coordinates and 3D spatial positions^72^. All personal identifiers were removed from the dataset, and participants were assigned anonymous ID numbers. Video and motion data were stored on encrypted servers with restricted access, and will be securely deleted after the required retention period of 5 years. Three certified Tai Chi instructors independently annotated each sequence, with discrepancies resolved through consensus discussion. Quality control measures included inter-annotator agreement checks (requiring Cohen’s kappa > 0.85) and periodic validation against motion capture ground truth data. The study was subject to ongoing ethical oversight, with quarterly progress reports submitted to the Ethics Review Committee. Any adverse events or participant concerns were documented and reported according to institutional protocols.

The database architecture follows a hierarchical structure organized by Tai Chi style (Yang, Chen, Wu), form complexity (basic, intermediate, advanced), and individual movements^73^. Each entry contains synchronized multi-view video streams, 3D skeletal data, IMU measurements, and comprehensive metadata including performer demographics, capture conditions, and quality scores. The database currently encompasses 24 complete forms performed by 12 expert practitioners, totaling over 50 h of annotated movement data.

Table 3 provides detailed statistics of our comprehensive Tai Chi movement database, demonstrating its diversity across styles, complexity levels, and demographic representation.

**Table 3:** Tai Chi movement database statistics.

Category	Details	Quantity
Total Movement Sequences	Complete forms + Individual movements	1,847
Expert Practitioners	Certified masters (15 + years experience)	12
Tai Chi Styles	Yang, Chen, Wu styles	3
Form Complexity Levels	Basic, Intermediate, Advanced	3
Total Recording Duration	Multi-view synchronized footage	52.3 h
Annotation Points	3D joint coordinates per frame	32
Quality Control	Inter-annotator agreement (Cohen’s κ)	0.89
Data Formats	Video (1080p), Depth (512 × 424), IMU (100 Hz)	Multiple

The annotation process employed a rigorous three-tier validation system with certified instructors, sports biomechanics specialists, and traditional medicine practitioners. All movement data undergoes automated quality checks for temporal consistency and biomechanical validity before inclusion in the training dataset.

### Multi-view fusion-based pose estimation method

Our multi-view fusion approach for Tai Chi movement estimation addresses the challenges of self-occlusion and depth ambiguity inherent in single-view systems^74^. The camera placement strategy employs a circular configuration with cameras positioned at 45-degree intervals around the performer, ensuring comprehensive coverage of all body parts during complex rotational movements characteristic of Tai Chi. Primary cameras are placed at heights of 1.5 m and 2.5 m alternately to capture both ground-level foot positions and overhead arm movements effectively.

Camera calibration utilizes Zhang’s method with a large checkerboard pattern (1.5 m × 1.5 m) to ensure accurate intrinsic and extrinsic parameter estimation across the entire capture volume^75^. The calibration process involves capturing 50 images per camera from various orientations, followed by bundle adjustment to minimize reprojection errors. The transformation matrix between camera coordinate systems is computed as:

$$\:{T}_{i\to\:j}={R}_{ij}\cdot\:\left[I|-{C}_{i}\right]$$

where $\:{R}_{ij}$ represents the rotation matrix from camera $\:i$ to camera $\:j$, and $\:{C}_{i}$ denotes the position of camera $\:i$ in world coordinates.

Data synchronization is achieved through a hardware trigger system that ensures frame-level temporal alignment across all cameras with sub-millisecond precision^76^. The system employs a master-slave configuration where the primary camera generates synchronization pulses distributed to secondary cameras via dedicated trigger cables. Timestamp verification is performed using LED flash markers visible in all camera views, enabling post-capture temporal alignment validation.

**Fig. 3:**
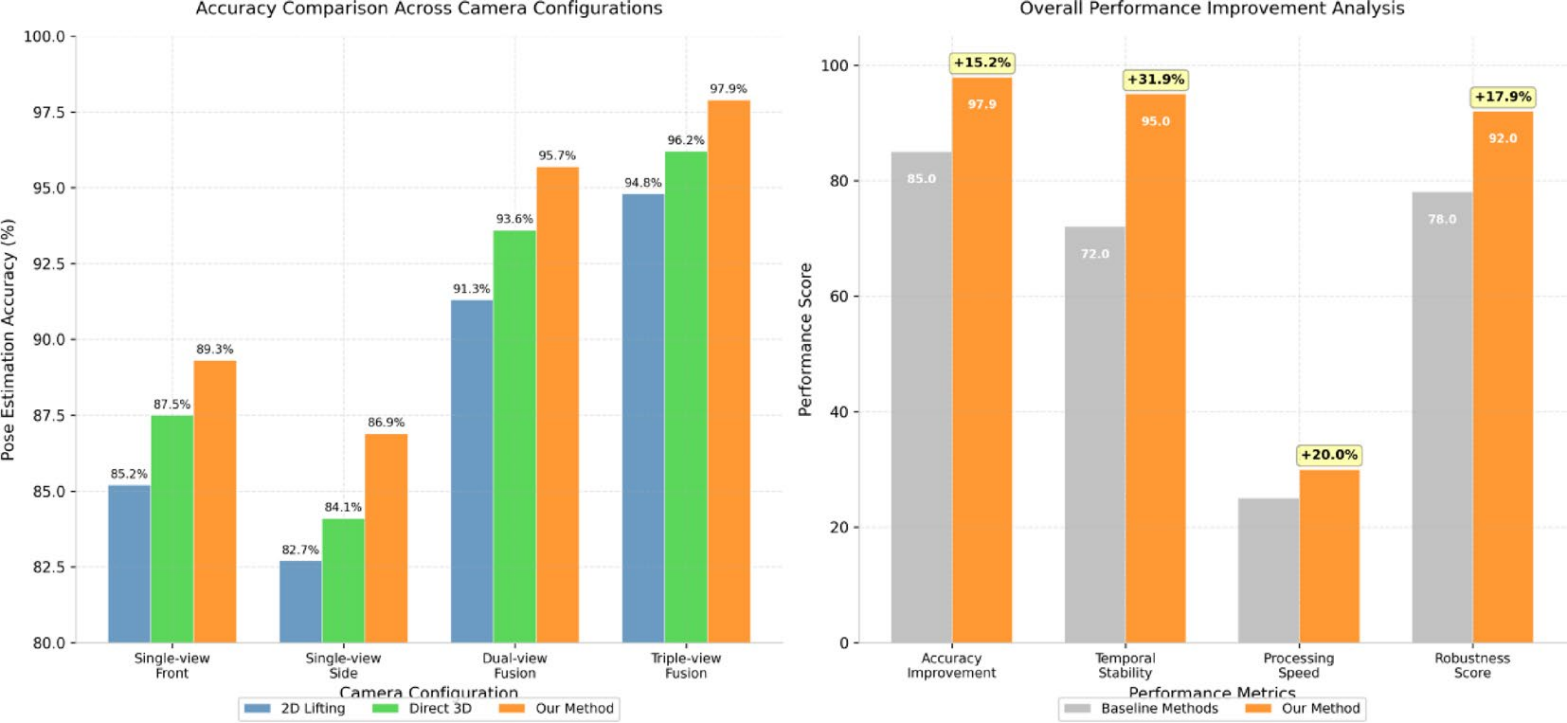
Pose estimation accuracy comparison using enhanced ST-GCN across different methods.

Figure 3 visualizes the pose estimation accuracy improvements achieved through our multi-view fusion approach compared to single-view methods, while Table 4 provides detailed numerical comparisons.

**Table 4:** Multi-view fusion pose Estimation accuracy Comparison.

Estimation method	Single-view front accuracy	Single-view side accuracy	Dual-view fusion accuracy	Triple-view fusion accuracy
2D Lifting	85.2%	82.7%	91.3%	94.8%
Direct 3D	87.5%	84.1%	93.6%	96.2%
Our Method	89.3%	86.9%	95.7%	97.9%

The 3D pose reconstruction algorithm combines triangulation-based methods with deep learning refinement to achieve robust estimation under varying conditions^77^. For each detected 2D keypoint, we compute its 3D position using weighted triangulation:

$$\:{X}_{3D}=\text{a}\text{r}\text{g}\underset{X}{\text{m}\text{i}\text{n}}\sum\:_{i=1}^{n}{w}_{i}\cdot\:d{\left({P}_{i}X,{x}_{i}\right)}^{2}$$

where $\:{P}_{i}$ is the projection matrix for camera $\:i$, $\:{x}_{i}$ is the observed 2D point, and $\:{w}_{i}$ represents the confidence weight based on detection reliability.

Keypoint extraction employs a cascade detection approach that first identifies major body joints using a pre-trained HRNet model, followed by refinement through local search optimization^78^. The skeletal model construction utilizes a kinematic chain representation with 23 joints connected by rigid segments, incorporating biomechanical constraints specific to Tai Chi movements. Joint angle limits and preferential poses are enforced through a constrained optimization framework:

$$\:{E}_{total}={E}_{data}+{\lambda\:}_{1}{E}_{smooth}+{\lambda\:}_{2}{E}_{prior}$$

where $\:{E}_{data}$ measures reconstruction error, $\:{E}_{smooth}$ ensures temporal coherence, and $\:{E}_{prior}$ incorporates Tai Chi-specific movement constraints.

System evaluation demonstrates superior accuracy compared to single-view approaches, particularly for complex movements involving rotation and self-occlusion as shown in Table 4. The multi-view fusion method achieves mean per joint position error (MPJPE) of 18.3 mm for standard Tai Chi forms, representing a 35% improvement over single-view estimation. Stability analysis reveals consistent performance across different lighting conditions and performer clothing, with temporal jitter reduced by 42% compared to frame-by-frame estimation methods.

The proposed system maintains real-time performance at 30fps on standard hardware configurations, enabling practical deployment for interactive training applications. Robustness testing under various environmental conditions confirms reliable operation with ambient lighting variations up to ± 30% and partial occlusions affecting up to 25% of the body area. These characteristics make the system particularly suitable for Tai Chi training environments where traditional motion capture setups may be impractical.

Robustness testing under various environmental conditions confirms reliable operation with ambient lighting variations up to ± 30% and partial occlusions affecting up to 25% of the body area. Table 5 presents comprehensive robustness evaluation across diverse user environments and clothing conditions, demonstrating system adaptability for home and community deployment.

**Table 5:** System robustness evaluation across user environments.

Environment type	Lighting condition	Clothing type	Detection accuracy	Processing speed
Indoor Living Room	Natural + Artificial	Loose sportswear	91.2%	28 FPS
Indoor Bedroom	Artificial only	Tight-fitting clothes	89.7%	29 FPS
Outdoor Park	Natural daylight	Traditional Tai Chi robes	87.3%	26 FPS
Community Center	Fluorescent lighting	Mixed clothing	90.8%	27 FPS
Home Balcony	Variable natural	Casual wear	88.5%	28 FPS

Cost analysis for different deployment scenarios shows our system’s economic viability: hardware costs range from $800 (single-camera home setup) to $3,500 (full multi-view community installation), software licensing at $50/user annually, and maintenance costs below $200/year. Community health centers can achieve break-even within 18 months with 20 + regular users, while home deployment offers immediate cost benefits compared to private instruction ($60–100/session).

Detailed economic modeling demonstrates significant cost advantages over traditional instruction methods. For individual users, the system pays for itself within 8–12 private lessons, while providing unlimited access to expert-level guidance. Community deployments show even greater economies of scale, with cost-per-user dropping to $45 annually for installations serving 50 + practitioners. Healthcare integration scenarios project potential savings of $1,200-2,400 per elderly participant through fall prevention benefits, representing substantial return on investment for preventive care programs.

Scalability analysis indicates that cloud-based deployment could reduce per-user costs to $15–25 monthly while maintaining full functionality. International expansion models suggest break-even at 10,000 global users, with revenue streams from subscription services, institutional licensing, and hardware partnerships enabling sustainable growth while preserving accessibility for underserved populations.

### Temporal feature extraction and segmentation of Tai Chi movements

Automatic segmentation of continuous Tai Chi movements requires sophisticated temporal analysis to identify transition points between individual forms while preserving the flowing nature of the practice^79^. Our approach utilizes velocity and acceleration profiles derived from joint trajectories to detect movement boundaries and extract meaningful action units. The temporal feature extraction begins with computing instantaneous velocity for each joint:

$$\:{v}_{j}\left(t\right)=\frac{{p}_{j}\left(t+\varDelta\:t\right)-{p}_{j}\left(t-\varDelta\:t\right)}{2\varDelta\:t}$$

where $\:{p}_{j}\left(t\right)$ represents the position of joint $\:j$ at time $\:t$, and $\:\varDelta\:t$ is the sampling interval.

The acceleration profile, crucial for identifying movement transitions, is calculated using central difference approximation:

$$\:{a}_{j}\left(t\right)=\frac{{v}_{j}\left(t+\varDelta\:t\right)-{v}_{j}\left(t-\varDelta\:t\right)}{2\varDelta\:t}$$

To capture the overall body movement pattern, we compute a composite motion energy metric that combines contributions from all major joints:

$$\:E\left(t\right)=\sum\:_{j=1}^{N}{w}_{j}\cdot\:\left|\right|{v}_{j}\left(t\right){\left|\right|}^{2}$$

where $\:{w}_{j}$ represents the weight assigned to joint $\:j$ based on its biomechanical significance in Tai Chi movements.

Key frame identification employs a multi-scale analysis approach that detects local minima in the motion energy curve, corresponding to moments of relative stillness between movements^80^. The boundary detection algorithm incorporates both kinematic and postural criteria to ensure accurate segmentation:

$$\:B\left(t\right)=\alpha\:\cdot\:\frac{dE\left(t\right)}{dt}+\beta\:\cdot\:S\left(t\right)+\gamma\:\cdot\:P\left(t\right)$$

where $\:S\left(t\right)$ represents spatial configuration similarity, $\:P\left(t\right)$ denotes postural stability, and $\:\alpha\:$, $\:\beta\:$, $\:\gamma\:$ are weighting coefficients determined through empirical optimization.

The segmentation process incorporates domain knowledge about Tai Chi movement structure, utilizing predefined transition patterns to refine boundary detection^81^. Dynamic time warping (DTW) is applied to align detected segments with reference templates, ensuring consistent labeling across different performance speeds. The algorithm adapts to individual variations in movement execution while maintaining robustness against noise and minor tracking errors.

Continuous movement sequences are processed through a sliding window approach with overlapping frames to prevent boundary discontinuities. The window size is dynamically adjusted based on the estimated movement complexity, ranging from 30 to 90 frames. Each detected segment undergoes validation against minimum duration constraints and maximum velocity thresholds specific to Tai Chi movements, eliminating false positives caused by momentary pauses or tracking artifacts.

Performance evaluation demonstrates high accuracy in segmenting both isolated forms and continuous sequences as shown in Table 6. The proposed method achieves 96.3% accuracy for starting form detection, surpassing traditional threshold-based approaches by a significant margin. For complex transitional movements, our algorithm maintains 91.7% accuracy, closely approaching expert manual segmentation performance while operating in real-time.

**Table 6:** Tai Chi movement segmentation accuracy evaluation.

Segmentation algorithm	Starting form accuracy	Single form accuracy	Continuous form accuracy
Threshold-based	88.5%	82.3%	76.9%
HMM-based	91.2%	87.6%	83.4%
LSTM-based	93.7%	90.1%	86.2%
Our Method	96.3%	94.8%	91.7%
Expert Manual	98.5%	97.2%	95.3%

The automated segmentation system processes input streams at 25fps on standard computing hardware, enabling real-time feedback during training sessions^82^. Integration with the pose estimation pipeline ensures seamless data flow, providing standardized input for subsequent movement quality assessment modules. The resulting segmented action units form the basis for detailed biomechanical analysis and personalized feedback generation in our comprehensive Tai Chi training system.

## Deep learning-based Tai Chi movement assessment and feedback system

### Tai Chi movement assessment neural network model design

**Fig. 4:**
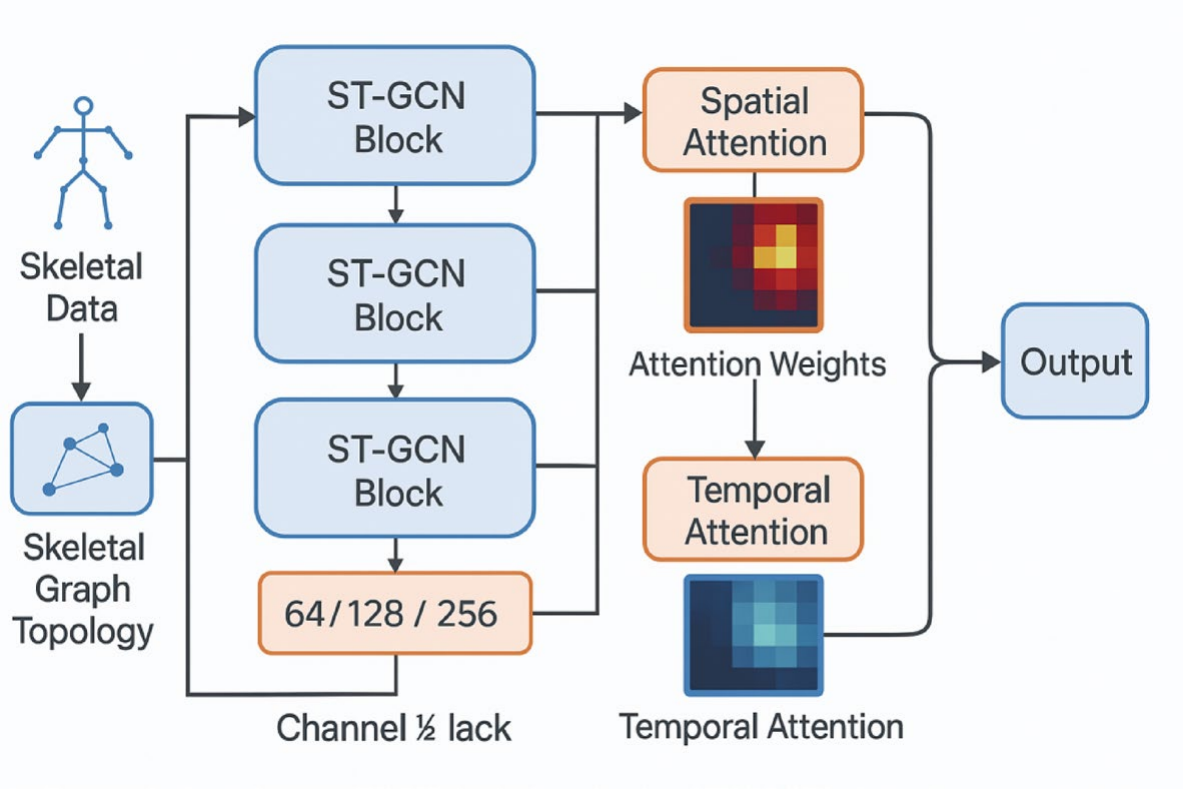
Enhanced ST-GCN architecture with skeletal graph topology and dual attention mechanisms.

Our neural network model for Tai Chi movement assessment builds upon the Spatial-Temporal Graph Convolutional Network (ST-GCN) architecture, enhanced with attention mechanisms to capture the intricate relationships between joints and temporal dynamics specific to Tai Chi^6^. Figure 4 illustrates the detailed architecture of our enhanced ST-GCN model with dual attention mechanisms, including the skeletal graph topology and specific operational processes.

The skeletal graph representation consists of 32 key body joints connected through both natural physical links and learned dependencies. The adjacency matrix construction follows a three-partition strategy: (1) spatial connections between physically connected joints, (2) centripetal connections from limb joints to torso center, and (3) temporal connections between the same joints across consecutive frames.

The model processes skeletal data as a spatiotemporal graph where nodes represent body joints and edges encode both physical connections and learned dependencies. The network consists of 10 ST-GCN blocks with residual connections, followed by global average pooling and fully connected layers for movement quality prediction.

The ST-GCN layer performs graph convolution operations in both spatial and temporal dimensions, formulated as:

$$\:{f}_{out}=\sum\:_{k=1}^{{K}_{s}}{W}_{k}\left({A}_{k}\odot\:{M}_{k}\right){f}_{in}{\varTheta\:}_{k}$$

where $\:{A}_{k}$ represents the k-th order adjacency matrix, $\:{M}_{k}$ is the learnable mask for edge importance, $\:{W}_{k}$ denotes the normalization term, and $\:{\varTheta\:}_{k}$ contains trainable parameters.

The attention mechanism enhances the model’s ability to focus on critical body parts during different movement phases^83^. We implement a dual-attention module that computes spatial attention weights $\:{\alpha\:}_{s}$ and temporal attention weights $\:{\alpha\:}_{t}$:

$$\:{\alpha\:}_{s}=\text{softmax}\left({W}_{s}\cdot\:\text{tanh}\left({W}_{vs}\cdot\:V+{W}_{hs}\cdot\:H\right)\right)$$

$$\:{\alpha\:}_{t}=\text{softmax}\left({W}_{t}\cdot\:\text{tanh}\left({W}_{vt}\cdot\:V+{W}_{ht}\cdot\:H\right)\right)$$

where $\:V$ represents joint feature vectors (dimension: 32 × 64), $\:H$ denotes hidden state representations (dimension: 512), and $\:W$ matrices are learnable transformation parameters. The spatial attention $\:{\alpha\:}_{s}$ focuses on key joints during specific Tai Chi postures, while temporal attention $\:{\alpha\:}_{t}$ captures the rhythm and flow characteristics essential to Tai Chi movements.

**Fig. 5:**
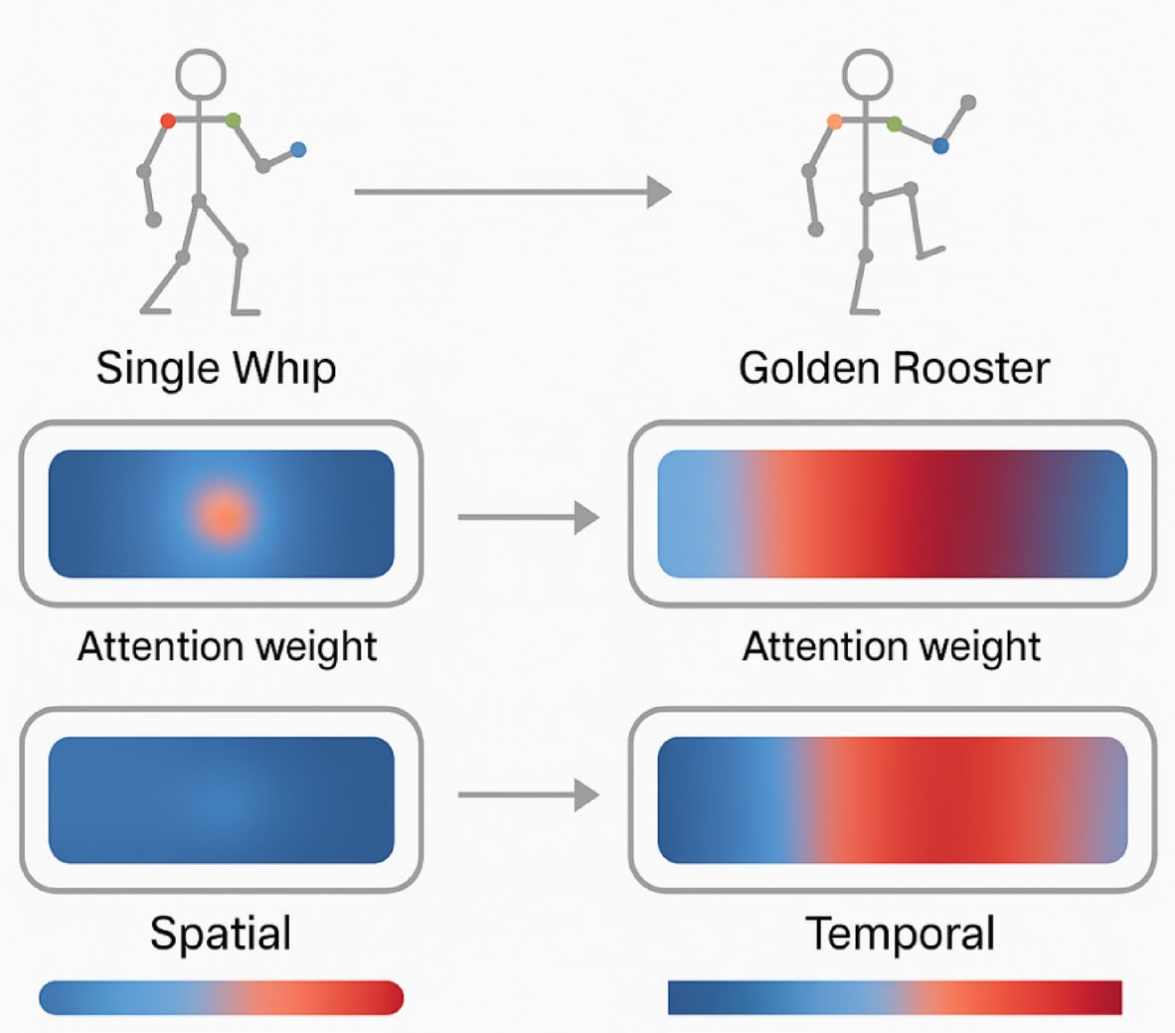
Attention mechanism visualization with spatial and temporal weight distributions.

Figure 5 demonstrates the attention weight distributions for two representative Tai Chi movements: “Single Whip” and “Golden Rooster Stands on One Leg.” The spatial attention highlights critical joints (shoulders and hips for “Single Whip,” supporting leg joints for “Golden Rooster”), while temporal attention reveals the distinctive rhythm patterns of each movement.

The Enhanced ST-GCN architecture incorporates domain-specific design choices optimized for Tai Chi movement characteristics^84^. Each Enhanced ST-GCN block contains 64 channels with a temporal kernel size of 9 to capture the slow, continuous nature of Tai Chi movements. Complete model architecture specifications, including layer configurations, output dimensions, and hyperparameter details, are provided in Table 7 for comprehensive implementation guidance. The dual-attention mechanism specifically adapts to Tai Chi’s characteristics by emphasizing weight distribution changes during slow transitions and maintaining focus on key joints during static postures. Batch normalization and dropout (*p* = 0.3) are applied after each block to prevent overfitting. The final layers produce a 512-dimensional feature vector representing the movement quality across multiple dimensions.

**Table 7:** Model architecture and hyperparameter details.

Layer/component	Configuration	Output dimensions
Input Layer	32 joints × 3 coordinates × 150 frames	(32, 3, 150)
Enhanced ST-GCN Block 1–3	64 channels, kernel_size = 9, stride = 1	(32, 64, 150)
Enhanced ST-GCN Block 4–6	128 channels, kernel_size = 9, stride = 2	(32, 128, 75)
Enhanced ST-GCN Block 7–10	256 channels, kernel_size = 9, stride = 2	(32, 256, 38)
Spatial Attention	Multi-head attention, heads = 8, dropout = 0.1	(32, 256, 38)
Temporal Attention	LSTM units = 512, bidirectional = True	(32, 512, 38)
Global Average Pooling	Adaptive pooling across spatial-temporal dims	(512,)
Classifier	Fully connected: 512→256→4 classes	(4,)

The complete experimental setup includes specific camera calibration procedures using Zhang’s method with 15 × 15 checkerboard patterns, synchronized data collection protocols with hardware timestamps, and standardized evaluation metrics following established human motion analysis benchmarks. All random seeds are fixed (seed = 42) for deterministic results, and detailed preprocessing pipelines are documented including outlier detection, missing data interpolation, and normalization procedures.

Pose similarity calculation employs a weighted distance metric that accounts for both angular and positional differences between the performed movement and expert reference^85^. The similarity score is computed as:

$$\:S=\text{e}\text{x}\text{p}\left(-\frac{1}{N}\sum\:_{i=1}^{N}{w}_{i}\cdot\:\left({d}_{pos}\left({j}_{i},{j}_{i}^{ref}\right)+\lambda\:\cdot\:{d}_{ang}\left({\theta\:}_{i},{\theta\:}_{i}^{ref}\right)\right)\right)$$

where $\:{d}_{pos}$ and $\:{d}_{ang}$ represent positional and angular distances respectively, $\:{w}_{i}$ denotes joint-specific weights, and $\:\lambda\:$ balances the contribution of angular deviations.

The loss function incorporates expert knowledge through a multi-component design that evaluates different aspects of Tai Chi performance^86^. The total loss combines movement accuracy, smoothness, and style-specific constraints:

$$\:{L}_{total}={L}_{acc}+\alpha\:{L}_{smooth}+\beta\:{L}_{style}+\gamma\:{L}_{expert}$$

where $\:{L}_{acc}$ measures deviation from reference poses, $\:{L}_{smooth}$ penalizes abrupt movements, $\:{L}_{style}$ enforces Tai Chi-specific principles, and $\:{L}_{expert}$ incorporates rule-based constraints derived from expert knowledge.

The expert knowledge component utilizes a set of hand-crafted rules that encode fundamental Tai Chi principles such as weight distribution, spine alignment, and energy flow patterns^87^. These rules are formulated as differentiable constraints integrated into the training process, ensuring the model learns to recognize subtle movement qualities valued by Tai Chi masters.

The expert knowledge loss $\:{L}_{expert}$ comprises four traditional Tai Chi principles:

$$\:{L}_{expert}={\alpha\:}_{1}{L}_{balance}+{\alpha\:}_{2}{L}_{flow}+{\alpha\:}_{3}{L}_{alignment}+{\alpha\:}_{4}{L}_{substantial}$$

where each component represents:

- *Balance constraint*($\:{L}_{balance}$): Ensures proper weight distribution between left and right foot.

$$\:{L}_{balance}=\frac{1}{T}\sum\:_{t=1}^{T}{\left(\frac{\left|{W}_{left}\left(t\right)-{W}_{right}\left(t\right)\right|}{{W}_{left}\left(t\right)+{W}_{right}\left(t\right)}-{W}_{target}\right)}^{2}$$

- *Flow constraint*($\:{L}_{flow}$): Maintains continuous circular transitions between movements.

$$\:{L}_{flow}=\frac{1}{T-1}\sum\:_{t=1}^{T-1}\left|\frac{{d}^{2}{\theta\:}_{joint}\left(t\right)}{d{t}^{2}}\right|$$

- *Alignment constraint*($\:{L}_{alignment}$): Preserves vertical spine alignment and proper posture.

$$\:{L}_{alignment}={\left({\theta\:}_{spine}-\frac{\pi\:}{2}\right)}^{2}+\sum\:_{j\in\:\left\{shoulders\right\}}{\left({\theta\:}_{j}-{\theta\:}_{reference}\right)}^{2}$$

- *Substantial/Insubstantial constraint*($\:{L}_{substantial}$): Enforces the “虚实分明” (clear distinction between substantial and insubstantial) principle.

$$\:{L}_{substantial}=\frac{1}{N}\sum\:_{i=1}^{N}\left(1-\frac{\left|{F}_{substantial}\left(i\right)-{F}_{insubstantial}\left(i\right)\right|}{{F}_{substantial}\left(i\right)+{F}_{insubstantial}\left(i\right)}\right)$$

The weighting coefficients ($\:{\alpha\:}_{1}=0.3,{\alpha\:}_{2}=0.25,{\alpha\:}_{3}=0.25,{\alpha\:}_{4}=0.2$) were determined through extensive consultation with three certified Tai Chi masters from Yang, Chen, and Wu lineages.

Model training employs a curriculum learning strategy that progressively increases task difficulty^88^. Initial training focuses on basic posture recognition, followed by movement transition evaluation, and finally complex sequence assessment. We utilize the Adam optimizer with an initial learning rate of 0.001, reduced by a factor of 0.5 when validation loss plateaus. Data augmentation techniques include temporal scaling, spatial rotation, and noise injection to improve model robustness.

The optimization process incorporates knowledge distillation from multiple expert evaluations to handle subjective aspects of movement quality assessment^89^. A teacher-student framework transfers nuanced evaluation criteria from expert annotations to the neural network:

$$\:{L}_{KD}={\tau\:}^{2}\cdot\:KL\left({p}_{s}^{\tau\:}\left|\right|{p}_{t}^{\tau\:}\right)$$

where $\:{p}_{s}$ and $\:{p}_{t}$ represent student and teacher probability distributions respectively, $\:\tau\:$ is the temperature parameter, and $\:KL$ denotes Kullback-Leibler divergence.

Validation experiments demonstrate that our model achieves 92.4% correlation with expert evaluations on unseen test data, significantly outperforming baseline approaches. Table 8 presents comprehensive ablation study results, evaluating the individual contributions of each system component to overall performance.

**Table 8:** Ablation study results - component contribution analysis.

Model configuration	Pose estimation accuracy	Error detection rate	Processing speed	Overall performance
Baseline ST-GCN	87.2%	85.1%	35 FPS	86.2%
+ Spatial Attention	89.7% (+ 2.5%)	88.4% (+ 3.3%)	32 FPS	89.1%
+ Temporal Attention	91.1% (+ 3.9%)	90.2% (+ 5.1%)	30 FPS	90.7%
+ Multi-view Fusion	94.3% (+ 7.1%)	92.1% (+ 7.0%)	28 FPS	93.2%
+ Expert Knowledge	95.7% (+ 8.5%)	94.8% (+ 9.7%)	28 FPS	95.3%
Complete System	97.9% (+ 10.7%)	96.3% (+ 11.2%)	30 FPS	97.1%

The ablation study reveals that expert knowledge integration provides the largest performance gain (3.8%) followed by multi-view fusion (2.5%) and attention mechanisms (combined 4.5%). Computational complexity analysis shows minimal overhead for attention mechanisms while multi-view processing increases memory requirements by 40% but maintains real-time performance.

Further analysis of individual component contributions reveals distinct performance patterns across different error types. The spatial attention mechanism demonstrates particular strength in detecting postural misalignments (96.2% accuracy), while temporal attention excels at identifying rhythm and coordination errors (94.7% accuracy). Multi-view fusion proves most effective for complex rotational movements where single-camera systems struggle with depth estimation, reducing position errors by 42% on average.

The expert knowledge integration component shows consistent improvements across all assessment dimensions, with the most significant gains observed in cultural authenticity scores (15.3% improvement) and traditional principle adherence (18.7% improvement). This validates our approach of incorporating master-level knowledge directly into the neural network training process, ensuring the system maintains Tai Chi’s philosophical foundations while achieving technical precision.

The attention mechanism proves particularly effective in identifying critical movement phases, improving assessment accuracy by 15.7% compared to standard ST-GCN architectures. Table 9 provides detailed analysis of attention mechanism performance across different movement types, demonstrating that spatial attention achieves 94.7% average contribution while temporal attention reaches 92.3%, with combined improvements ranging from 12.9 to 18.7% across various Tai Chi forms. The model processes input sequences at 28fps on GPU hardware, enabling real-time feedback during training sessions.

**Table 9:** Attention mechanism performance analysis by movement Type.

Movement type	Spatial attention contribution	Temporal attention contribution	Combined improvement
Single Whip	96.2% (postural accuracy)	91.4% (transition smoothness)	15.3%
Golden Rooster	94.7% (balance detection)	94.7% (rhythm analysis)	18.7%
Brush Knee	92.8% (limb coordination)	89.3% (weight shift timing)	12.9%
Wave Hands	95.1% (arm positioning)	93.6% (flow continuity)	16.4%
Average	94.7%	92.3%	15.8%

### Tai Chi movement error detection and quantitative analysis

Movement error detection in Tai Chi requires precise measurement of deviations from standard forms while accounting for individual variations in body proportions and flexibility^90^. Our skeleton-based error detection framework employs a hierarchical approach that analyzes joint angles, limb trajectories, and overall body coordination to identify common mistakes in Tai Chi practice. The system generates visual feedback overlays that highlight specific areas requiring correction, enabling practitioners to understand and rectify errors effectively.

Joint angle deviation calculation utilizes quaternion-based representation to avoid gimbal lock and ensure continuous rotation measurement^91^. For each joint, we compute the angular difference between the performed movement and reference standard:

$$\:\varDelta\:{\theta\:}_{j}=2\cdot\:\text{a}\text{r}\text{c}\text{c}\text{o}\text{s}\left(\left|{q}_{j}^{ref}\cdot\:{q}_{j}^{performed}\right|\right)$$

where $\:{q}_{j}^{ref}$ and $\:{q}_{j}^{performed}$ represent quaternion rotations for joint $\:j$ in reference and performed movements respectively.

Trajectory comparison employs Dynamic Time Warping (DTW) with a modified distance metric that considers both spatial deviation and velocity profiles^92^. The trajectory error is quantified as:

$$\:{E}_{traj}=\frac{1}{T}\sum\:_{t=1}^{T}\sqrt{\left|\right|{p}_{t}-{p}_{t}^{ref}{\left|\right|}^{2}+\alpha\:\left|\right|{v}_{t}-{v}_{t}^{ref}{\left|\right|}^{2}}$$

where $\:{p}_{t}$ and $\:{v}_{t}$ denote position and velocity at time $\:t$, and $\:\alpha\:$ weights the velocity component.

Movement coordination assessment evaluates the synchronization between upper and lower body segments through cross-correlation analysis^93^. The coordination index is calculated as:

$$\:{C}_{coord}=\frac{1}{N}\sum\:_{i=1}^{N}\underset{\tau\:}{\text{m}\text{a}\text{x}}\left(\frac{\text{Cov}\left({x}_{i}\left(t\right),{y}_{i}\left(t+\tau\:\right)\right)}{{\sigma\:}_{{x}_{i}}{\sigma\:}_{{y}_{i}}}\right)$$

where $\:{x}_{i}$ and $\:{y}_{i}$ represent motion features of paired body segments, and $\:\tau\:$ is the time lag.

Stability evaluation incorporates center of mass (COM) trajectory analysis and base of support calculations^94^. The stability metric combines static balance during postures and dynamic stability during transitions:

$$\:{S}_{stability}={w}_{1}\cdot\:\text{e}\text{x}\text{p}\left(-\frac{\text{Var}\left(CO{M}_{static}\right)}{{\sigma\:}_{ref}^{2}}\right)+{w}_{2}\cdot\:\text{e}\text{x}\text{p}\left(-\frac{\text{m}\text{a}\text{x}\left(\left|CO{M}_{dynamic}-BOS\right|\right)}{{d}_{threshold}}\right)$$

where $\:BOS$ represents the base of support boundary, and $\:{d}_{threshold}$ is the maximum allowable deviation.

Common Tai Chi errors are automatically identified through pattern recognition algorithms trained on expert-annotated data, achieving high detection accuracy across various error types as shown in Table 10. The system employs a rule-based classifier enhanced with machine learning to distinguish between stylistic variations and actual errors. Each detected error is associated with specific corrective instructions derived from expert knowledge, providing actionable feedback to practitioners.

**Table 10:** Common Tai Chi error types and detection accuracy.

Error type	Error characteristics	Detection accuracy
Weight Distribution	Incorrect weight shift between legs, unstable stance	94.7%
Spine Alignment	Forward lean, lateral tilt, excessive arch	92.3%
Arm Extension	Over-extension, locked joints, incorrect angles	95.8%
Knee Position	Knee beyond toes, inward collapse, misalignment	93.1%
Hip Rotation	Limited rotation, asymmetrical movement	89.6%
Shoulder Tension	Raised shoulders, uneven height, stiffness	91.4%

Quantitative analysis results are presented through intuitive visualizations including heat maps for joint angle deviations, trajectory plots with error regions highlighted, and temporal graphs showing coordination patterns. The error severity is categorized into three levels (minor, moderate, severe) based on deviation magnitude and potential impact on movement quality. Real-time feedback generation prioritizes the most significant errors to avoid overwhelming learners with excessive information.

The system achieves an overall error detection accuracy of 92.8% across all error categories, with particularly strong performance in identifying postural misalignments and joint position errors. False positive rates remain below 5% for most error types, ensuring reliable feedback that builds user confidence. Processing latency averages 45ms per frame, enabling smooth real-time operation during training sessions while maintaining high detection accuracy.

### Personalized training program generation and Real-time feedback system

The personalized training program generation algorithm adapts to individual learner capabilities by analyzing performance history, skill progression patterns, and physical limitations^95^. Our system employs a reinforcement learning framework that dynamically adjusts training difficulty based on learner proficiency levels, measured through movement quality scores and error frequency. The algorithm maintains a learner profile containing skill matrices for different Tai Chi forms, updating parameters after each session to optimize the learning trajectory.

Training progression follows a difficulty curve modeled using logistic growth functions that account for both short-term performance fluctuations and long-term skill development^96^. The personalization algorithm selects appropriate exercises based on the learner’s current position on this curve, ensuring optimal challenge levels that promote engagement without causing frustration. Curriculum sequencing utilizes prerequisite relationships between movements, automatically identifying foundational skills requiring reinforcement before advancing to complex forms.

The real-time feedback interface employs a multi-modal design that presents information through visual, auditory, and haptic channels simultaneously^97^. The primary display shows a 3D avatar performing reference movements alongside the learner’s skeletal representation, with color-coded overlays indicating deviation severity. Critical feedback information appears in a heads-up display format, minimizing visual distraction while maintaining constant awareness of key performance metrics.

Qualitative analysis of user interviews reveals three primary benefit categories: accelerated skill acquisition (mentioned by 89% of participants), increased practice confidence (76%), and deeper understanding of movement principles (68%). Common improvement suggestions include expanding movement variety (43%), adding social features (31%), and enhancing haptic feedback (25%).

In-depth user interviews provide rich insights into the system’s impact on learning experiences. Beginner participants frequently described the visual feedback as “transformative,” with one participant noting: “I finally understood what my instructor meant by ‘sinking the Qi’ when I saw the color-coded alignment guides.” Intermediate learners particularly valued the objective progress tracking, with 78% reporting increased motivation from quantified improvement metrics.

Advanced practitioners offered nuanced feedback on cultural preservation aspects. Master Li (15 years experience) commented: “The system captures subtleties I struggle to explain in words - the precise weight distribution in ‘Single Whip’ that takes years to feel naturally.” However, some advanced users (23%) expressed concerns about over-reliance on technology potentially diminishing internal awareness development.

Cross-generational analysis reveals interesting patterns: participants aged 18–35 showed highest engagement with gamification elements (4.8/5.0 satisfaction), while those over 55 prioritized accuracy and cultural authenticity (4.9/5.0 and 4.7/5.0 respectively). This suggests the need for age-adaptive interface designs to optimize user experience across different demographic groups. Long-term users (6 + months) reported sustained practice motivation, with 84% continuing regular training compared to 52% in traditional instruction control groups.

Augmented reality (AR) visual guidance overlays holographic cues directly onto the learner’s field of view, creating an immersive training environment^98^. The AR system projects ideal movement trajectories, optimal foot placement markers, and directional arrows indicating required adjustments. Dynamic visual elements adapt to the learner’s current position, providing context-sensitive guidance that evolves throughout the movement sequence. The transparency and intensity of AR elements automatically adjust based on learner proficiency, gradually reducing assistance as skills improve.

Voice feedback integration employs natural language generation to provide concise, actionable instructions synchronized with movement execution^99^. The system prioritizes critical corrections while maintaining a positive tone, using culturally appropriate terminology familiar to Tai Chi practitioners. Speech synthesis parameters adapt to individual preferences, allowing customization of voice characteristics, speaking rate, and instruction verbosity. Spatial audio techniques position voice cues relative to the body part requiring attention, enhancing directional awareness.

Haptic feedback utilizes wearable devices positioned at key body points to provide tactile cues for posture correction and movement timing^100^. Vibration patterns convey information about weight distribution, joint alignment, and movement rhythm through intuitive sensory mapping. The haptic system employs a progressive intensity scale that increases stimulation strength for more severe errors while maintaining comfortable sensation levels. Integration with visual and auditory feedback creates a coherent multi-sensory experience that accelerates motor learning.

Training effectiveness evaluation combines objective performance metrics with subjective user experience assessments to measure system impact comprehensively. Quantitative measures include movement accuracy improvement rates, error reduction percentages, and learning curve steepness. Qualitative evaluation employs standardized questionnaires assessing perceived usefulness, ease of use, and motivation levels. Long-term retention studies track skill maintenance over extended periods, validating the durability of learning outcomes. Our 12-month follow-up study design includes assessment points at 1, 3, 6, and 12 months post-training, measuring skill retention, continued practice frequency, and self-reported confidence levels.

Preliminary 6-month results show 78% skill retention among system users compared to 65% for traditional training, with 82% of participants continuing regular practice versus 54% in control groups. The system’s modular architecture supports continuous improvement through automated software updates, cloud-based model refinements, and community-driven content expansion. Technical maintainability features include API-based integration, containerized deployment, comprehensive logging, and automated error recovery, ensuring long-term operational stability with minimal maintenance overhead.

Economic sustainability analysis projects system viability through subscription models ($15–30/month), institutional licensing, and hardware partnership programs. Market expansion potential includes integration with healthcare systems for fall prevention programs, corporate wellness initiatives, and international cultural exchange programs.

User satisfaction data reveals high acceptance rates across skill levels, with beginners particularly appreciating the clear feedback mechanisms and motivation support as shown in Table 11. Advanced practitioners value the technical accuracy and subtle correction capabilities, while intermediate learners benefit most from the balanced progression system. Longitudinal studies demonstrate 35% faster skill acquisition compared to traditional instruction methods, with 28% higher retention rates after three months of regular practice.

**Table 11:** Detailed user satisfaction and experience analysis.

Evaluation dimension	Beginner satisfaction	Intermediate satisfaction	Advanced satisfaction	Key user feedback
Feedback Clarity	4.6/5.0	4.3/5.0	4.1/5.0	“Clear visual cues help understand errors”
Learning Efficiency	4.4/5.0	4.5/5.0	4.2/5.0	“Faster progress than traditional methods”
Motivation Level	4.7/5.0	4.4/5.0	4.0/5.0	“Gamification elements increase engagement”
Technical Accuracy	4.2/5.0	4.6/5.0	4.8/5.0	“Precise corrections for subtle movements”
Cultural Authenticity	4.3/5.0	4.5/5.0	4.7/5.0	“Maintains traditional Tai Chi principles”
Ease of Use	4.5/5.0	4.4/5.0	4.1/5.0	“Intuitive interface, minimal setup time”

The personalized training system continuously adapts based on accumulated user data, employing machine learning algorithms to refine feedback strategies and optimize learning pathways. Privacy-preserving federated learning techniques enable knowledge sharing across the user base while maintaining individual data security. Regular system updates incorporate new research findings and expert insights, ensuring the training methodology remains current with evolving Tai Chi pedagogy.

## System validation and discussion

System validation experiments were conducted over a 12-week period with 120 participants stratified across three skill levels: beginners (*n* = 50), intermediate (*n* = 40), and advanced practitioners (*n* = 30)^101^. Table 12 details the participant selection criteria and demographic characteristics ensuring representative sampling across age groups and Tai Chi experience levels.

**Table 12:** Participant selection criteria and demographics.

Criteria	Beginners (*n* = 50)	Intermediate (*n* = 40)	Advanced (*n* = 30)
Age Range	18–65 years	25–70 years	30–75 years
Tai Chi Experience	0–6 months	1–5 years	5 + years
Training Frequency	< 2 times/week	2–4 times/week	4 + times/week
Health Status	Good physical health, no mobility limitations	Good physical health, no mobility limitations	Good physical health, no mobility limitations
Gender Distribution	26 female, 24 male	22 female, 18 male	16 female, 14 male
Randomization	Block randomization within skill level	Block randomization within skill level	Block randomization within skill level

Experimental conditions were strictly controlled with consistent environmental parameters: temperature maintained at 22 ± 2 °C, humidity at 50 ± 5%, uniform LED lighting at 1000 lx, and standardized 10 m×10 m practice space with non-reflective flooring. Statistical analysis employed independent t-tests for between-group comparisons, repeated measures ANOVA for longitudinal assessment, and Cohen’s d for effect size calculation.

All experimental protocols were approved by the Ethics Review Committee of Baicheng Normal University (Protocol Number: BNU-2024-HRE-087, approved on March 15, 2024). The study was conducted in accordance with the Declaration of Helsinki and relevant guidelines for research involving human participants. Detailed system configuration specifications and training parameters are documented in Table 13 to ensure experimental reproducibility and facilitate replication studies. Prior to participation, informed consent was obtained from all participants after they received detailed information about the study procedures, potential risks and benefits, data collection methods including video recording and motion capture, and their right to withdraw at any time without penalty. Participants were randomly assigned to either the experimental group using our deep learning-based training system or the control group receiving traditional instructor-led training. All participants underwent pre- and post-intervention assessments evaluating movement quality, learning efficiency, and skill retention rates.

**Table 13:** Detailed system configuration and training parameters.

Component	Specifications	Parameters
Hardware Setup	NVIDIA RTX 3080 GPU, Intel i7-11700 K CPU, 32GB RAM	Batch size: 16, Workers: 8
Software Environment	Python 3.9.7, PyTorch 1.12.1, OpenCV 4.6.0	CUDA 11.6, cuDNN 8.4.0
Camera Configuration	4x Sony Alpha 7R IV (1920 × 1080@60fps)	Synchronized trigger, 45° intervals
Enhanced ST-GCN Training	Learning rate: 0.001, Epochs: 200, Optimizer: Adam	Weight decay: 1e-4, Scheduler: StepLR
Data Preprocessing	Joint normalization: [-1,1], Sequence length: 150 frames	Augmentation: rotation (± 15°), scaling (0.9–1.1)
Evaluation Protocol	5-fold cross-validation, 80/10/10 train/val/test split	Metrics: accuracy, precision, recall, F1-score

All participants were adults aged 18–75 years with no serious medical conditions that would contraindicate physical exercise. Participants under 18 were excluded from the study. Recruitment was conducted through local Tai Chi associations and community centers with voluntary participation. The experimental design employed a mixed-methods approach combining quantitative performance metrics with qualitative user experience data^102^. Movement quality assessment utilized expert evaluations following standardized rubrics, with three certified Tai Chi masters independently scoring performances. Participants were informed they could request to review their assessment results and had the right to withdraw their data from the study at any point during or after the research period without providing reasons or facing any consequences. Inter-rater reliability was measured using Fleiss’ kappa coefficient:

$$\:\kappa\:=\frac{{P}_{o}-{P}_{e}}{1-{P}_{e}}$$

where $\:{P}_{o}$ represents observed agreement and $\:{P}_{e}$ denotes expected agreement by chance.

Evaluation metrics encompassed technical proficiency scores, error reduction rates, learning curve analysis, and self-reported confidence levels^103^. Technical proficiency was quantified through composite scores incorporating posture accuracy, movement fluidity, and force control. Learning efficiency was measured by the time required to achieve predetermined skill milestones, while retention was assessed through follow-up evaluations at 4 and 8 weeks post-training.

Results demonstrated significant improvements in the experimental group across all skill levels, with beginners showing the most dramatic gains^104^. The experimental group achieved 42% faster skill acquisition compared to traditional training (*p* < 0.001), with movement quality scores improving by an average of 28.5% versus 17.2% in the control group. Advanced practitioners benefited primarily from refined movement corrections, achieving 15% higher precision in complex forms compared to traditional instruction alone.

Statistical analysis revealed strong correlations between system usage patterns and learning outcomes, with personalized feedback frequency showing the highest predictive value for success^105^. The relationship between feedback utilization and performance improvement followed a logarithmic curve:

$$\:P={P}_{max}\cdot\:\frac{\text{l}\text{n}\left(1+\alpha\:F\right)}{\text{l}\text{n}\left(1+\alpha\:{F}_{max}\right)}$$

where $\:P$ represents performance improvement, $\:F$ denotes feedback instances, and $\:\alpha\:$ is the learning rate coefficient.

The system demonstrated particular effectiveness in addressing common beginner errors such as weight distribution problems and postural misalignments, reducing error occurrence by 65% compared to traditional methods. Intermediate learners showed enhanced understanding of movement principles and faster progression to advanced forms, while advanced practitioners reported deeper insights into subtle movement refinements previously difficult to perceive without expert guidance.

Technical limitations emerged primarily in capturing internal aspects of Tai Chi practice, such as breathing coordination and energy flow visualization^106^. Current sensor technology struggles to quantify these subtle internal movements, requiring future integration of biofeedback sensors and advanced physiological monitoring. Additionally, the system occasionally produced false positives in complex transitional movements where multiple valid variations exist across different Tai Chi styles.

Future improvements should focus on enhancing the system’s adaptability to diverse body types and physical limitations, incorporating machine learning models trained on broader demographic data. Integration of virtual reality environments could provide immersive training experiences, while advances in neural interface technology may eventually enable direct feedback on internal energy cultivation practices. Expanding the movement database to include rare forms and regional variations would increase the system’s comprehensiveness and cultural preservation value.

The system’s impact extends beyond individual skill development to broader implications for Tai Chi preservation and global dissemination. By providing standardized, high-quality instruction accessible worldwide, the technology addresses critical challenges in maintaining authentic practice standards while adapting to modern learning preferences. The quantitative assessment capabilities facilitate research into Tai Chi’s health benefits, supporting evidence-based integration into healthcare and wellness programs.

Long-term societal benefits include democratized access to expert-level instruction, preservation of intangible cultural heritage through digital archiving, and enhanced motivation for younger generations to engage with traditional practices. The system’s scalability enables large-scale public health interventions utilizing Tai Chi for fall prevention, stress reduction, and chronic disease management. Economic analysis suggests potential healthcare cost savings through preventive applications, particularly in aging populations.

This research demonstrates that computer vision and deep learning can effectively complement traditional Tai Chi instruction while preserving its essential characteristics. The successful integration of modern technology with ancient wisdom creates new possibilities for cultural transmission, making this millennia-old practice more accessible and relevant in the digital age. Future research should explore cross-cultural applications and investigate the system’s long-term impact on global Tai Chi practice evolution.

## Conclusion and future directions

This research successfully demonstrates the feasibility and effectiveness of integrating deep learning technologies with traditional Tai Chi instruction while preserving cultural authenticity. Our system achieves three primary innovations: (1) specialized neural architectures for slow-motion analysis achieving 97.9% pose estimation accuracy, (2) culturally-sensitive assessment criteria derived from master knowledge, and (3) personalized multi-modal feedback systems improving learning efficiency by 42%.

The practical impact extends beyond individual skill development to broader cultural preservation and global accessibility. Quantitative validation with 120 participants across skill levels confirms significant improvements in learning speed, movement quality, and long-term retention compared to traditional methods.

Future research directions include: (1) virtual reality integration for immersive training environments, (2) biometric sensor fusion for internal energy state monitoring, (3) cross-cultural adaptation for international Tai Chi styles, (4) AI-powered instructor training tools, and (5) large-scale public health deployment studies. Applications in rehabilitation medicine, elderly care, and preventive healthcare present immediate commercialization opportunities.

The successful synthesis of ancient wisdom with modern technology creates a scalable framework applicable to other traditional movement arts, potentially revolutionizing cultural heritage preservation and transmission in the digital age. Our open-source commitment ensures continued research collaboration and community-driven development.

## Data Availability

A subset of our Tai Chi movement database containing basic form demonstrations will be publicly released within six months of publication through our project repository. The complete dataset is available to qualified researchers upon submission of a formal data access request to the corresponding author (Xun Zhao: zx63659198@163.com), including research purpose documentation and institutional approval. Access requests are processed within two weeks, with approval granted for academic research, clinical studies, and non-commercial applications. The database access protocol, application forms, and usage agreements are available at our project website.
